# Expression of the C-Terminal Domain of Phospholipase Cβ3 Inhibits Signaling via Gαq-Coupled Receptors and Transient Receptor Potential Channels

**DOI:** 10.3390/ijms23179590

**Published:** 2022-08-24

**Authors:** Gerald Thiel, Oliver G. Rössler

**Affiliations:** Department of Medical Biochemistry and Molecular Biology, Saarland University, Building 44, 66421 Homburg, Germany

**Keywords:** Gαq-coupled designer receptor, ERK1/2, *m*-3M3FBS, phospholipase Cβ, TRPM3, TRPM8

## Abstract

Transient receptor potential (TRP) channels are cation channels that play a regulatory role in pain and thermosensation, insulin secretion, and neurotransmission. It has been proposed that activation of TRP channels requires phosphatidylinositol 4,5-bisphosphate, the major substrate for phospholipase C (PLC). We investigated whether inhibition of PLCβ has an impact on TRP channel signaling. A genetic approach was used to avoid off-target effects observed when using a pharmacological PLCβ inhibitor. In this study, we show that expression of PLCβ1ct and PLCβ3ct, truncated forms of PLCβ1 or PLCβ3 that contain the C-terminal membrane binding domains, almost completely blocked the signal transduction of a Gαq-coupled designer receptor, including the phosphorylation of ERK1/2. In contrast, expression of the helix-turn-helix motif (Hα1—Hα2) of the proximal C-terminal domain of PLCβ3 did not affect Gαq-coupled receptor signaling. PLCβ3ct expression impaired signaling of the TRP channels TRPM3 and TRPM8, stimulated with either prognenolone sulfate or icilin. Thus, the C-terminal domain of PLCβ3 interacts with plasma membrane targets, most likely phosphatidylinositol 4,5-bisphosphate, and in this way blocks the biological activation of TRPM3 and TRPM8, which require interaction with this phospholipid. PLCβ thus regulates TRPM3 and TRPM8 channels by masking phosphatidylinositol 4,5-bisphosphate with its C-terminal domain.

## 1. Introduction

TRP (transient receptor potential) channels are cation channels that have a similar modular structure, but differ substantially in their primary structures. TRP channels have six transmembrane regions and the ion pore has been located between transmembrane regions 5 and 6. Ca^2+^ ions and/or Na^+^ ions can flow into the cells from the extracellular environment through this pore, dependent on the TRP channel. TRP channels play an important role in the regulation of sensory pathways, thermosensation, pain sensation, mechanoperception, and perception of pungent chemicals. In addition, TRP channels have been described as being involved in the regulation of tumorigenesis, cell proliferation, insulin secretion, and neurotransmission [1]. TRP channels are embedded into the plasma membrane, and it has been proposed that channel activity is modulated by the plasma membrane phospholipid phosphatidylinositol 4,5-bisphosphate, which acts as a necessary cofactor for other ion channels [2]. Phosphatidylinositol 4,5-bisphosphate is a negatively charged phospholipid located at the cytoplasmic side of the plasma membrane and binds to ion channels via electrostatic interactions.

It has been reported that the biological activities of TRP channels TRPM3 and TRPM8 are regulated by phosphatidylinositol 4,5-bisphosphate [3,4,5,6,7]. TRPM3 channels are cation channels that respond to heat and the steroid pregnenolone sulfate [8,9]. Activation of TRPM3 has been linked to temperature and pain sensation, vascular smooth muscle contraction, gene transcription, insulin secretion, and tumorigenesis [10]. TRPM3 functions as a chemo- and thermosensor in the somatosensory system and, together with the TRPA1 and TRPV1 channels, regulates heat sensation [11,12]. TRPM3 activity is enhanced by phosphoinositides in cell-free inside-out patches, and inhibited by the expression of ci-VSP, a voltage-sensing phosphatase that catalyzes the removal of 5-phosphate from phosphoinositides [3,4].

TRPM8 channels mediate the influx of Ca^2+^ ions into the cells after stimulation with cooling agents (menthol, icilin, eucalyptol) and by reduced temperature [13]. TRPM8 channels are present in a subset of temperature-sensing dorsal root and trigeminal and ganglion neurons and function as cold nociceptors in these neurons, mediating nocifensive responses to noxious cold [14,15]. TRPM8 channel activity has been linked to a number of diseases, including migraines and cancer [13]. Expression of the phosphatase ci-VSP inhibited TRPM8-induced current, suggesting that the plasma membrane phosphatidylinositol 4,5-bisphosphate concentration is important for TRPM8 channel activity [4,5].

Phospholipase C (PLC) catalyzes the hydrolysis of phosphatidylinositol 4,5-bisphosphate, generating 1,4,5-triphosphate (IP_3_) and diacylgycerol (DAG). Thus, activation of PLC reduces the concentration of phosphatidylinositol 4,5-bisphosphate. Assuming that phosphatidylinositol 4,5-bisphosphate is required for TRPM3 and TRPM8 activity, PLC activation should negatively influence TRPM3 and TRPM8 channel activity. Accordingly, Gαq-coupled receptor activation of PLCβ has been shown to inhibit TRPM3 and TRM8 activities [3,4,6]. Furthermore, activation of TrkA receptors or PDGF receptors, which trigger an activation of PLCγ, resulted in inhibition of TRPM8 current [6,7].

Previous studies have measured the reduction of membrane-bound phosphatidylinositol 4,5-bisphosphate via an indirect assay based on the translocation of a fluorescently labeled PLCγ-derived PH domain from the plasma membrane to the cytoplasm. The use of this translocation assay has some pitfalls, as this PH domain does not bind specifically to phosphatidylinositol 4,5-bisphosphate but binds 20-fold more strongly to IP_3_ [2,16] and may act as an IP_3_ sponge that attenuates IP_3_ downstream signaling events. In addition, PLCγ-PH binding to the membrane can be disrupted by increased intracellular Ca^2+^ concentrations [17], as is the case after stimulation of Gαq-coupled receptors or TRP channels. Furthermore, by decorating the plasma membrane the GFP-tagged PH domain fusion proteins sequester their target and may interfere with the binding of other proteins to phosphatidylinositol 4,5-bisphosphate, disrupting intracellular signaling pathways downstream of TRP channels and producing off-target effects.

In this study, we did not attempt to affect the level of phosphatidylinositol 4,5-bisphosphate at the plasma membrane. Rather, we used a genetic tool to inhibit PLCβ activity. Expression of the C-terminal domain of either PLCβ1 or PLCβ3 almost completely inhibited Gαq-coupled receptor signaling. Under these circumstances, TRPM3 and TRPM8 intracellular signaling was impaired as well. The C-terminal domain of PLCβ harbors the primary membrane tagging site of the enzyme. We conclude that PLCβ and TRPM channels compete for the same target(s) at the plasma membrane, suggesting that membrane-bound PLCβ negatively affects TRPM3 and TRPM8 activities without altering the concentration of phosphatidylinositol 4,5-bisphosphate. Furthermore, we show that the compound *m*-3M3FBS, described as a pharmacological activator of PLC, does not trigger a signaling cascade comparable to that initiated by stimulating Gαq-coupled receptors. Nevertheless, treatment of the cells with *m*-3M3FBS effectively impaired TRPM3 and TRPM8 channel signaling.

## 2. Results

### 2.1. Expression of the C-Terminal Domain of Phospholipase Cβ3 Blocks Signaling Induced by Stimulation of a Gαq-Coupled Designer Receptor

**Figure 1:**
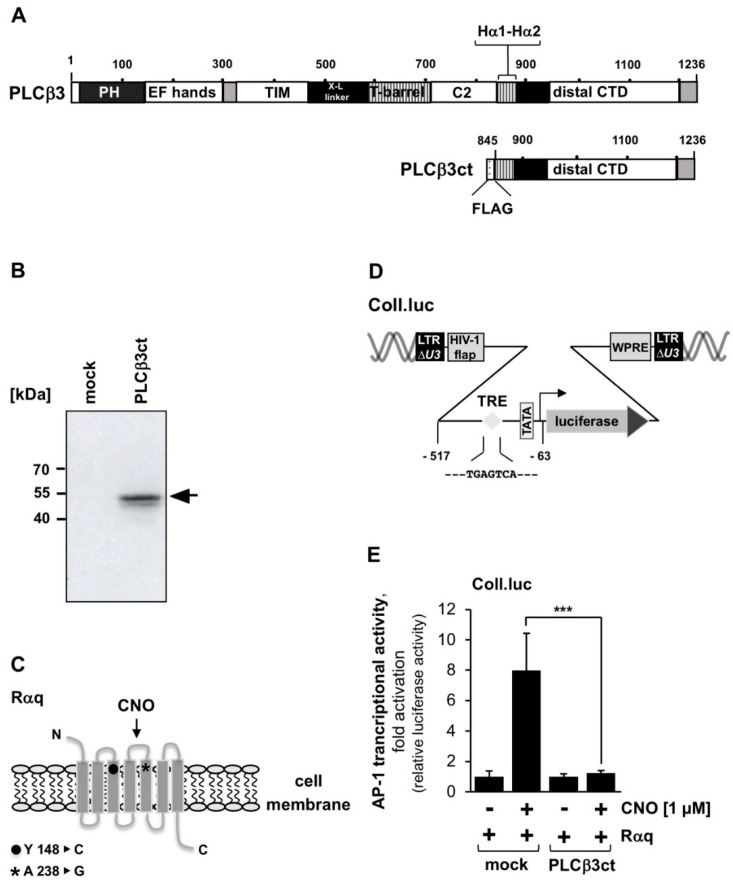
Expression of the C-terminal domain of phospholipase Cβ3 blocks signaling of Rαq, a Gαq-coupled designer receptor. (**A**) Multi-domain structure of phospholipase Cβ3 and the truncated mutant PLCβ3ct. PLCβ3 contains a pleckstrin homology (PH) domain, four tandem EF-hand repeats, the catalytic triose phosphate isomerase (TIM) barrel domain, the C2 domain, the helix-turn-helix motif (Hα1—Hα2), and a C-terminal domain (CTD). (**B**) Expression of PLCβ3ct mutant in HEK293 cells following infection of the cells with an PLCβ3ct-encoding lentivirus. Cells were infected with a lentivirus encoding either PLCβ3ct or β-galactosidase (mock). The Western blot was developed with an antibody against the FLAG-tag. kDa, molecular mass markers. (**C**) Modular structure of Rαq. The two-point mutations essential to change the M3 muscarinic acetylcholine receptor into a Gαq-coupled, CNO-sensitive designer receptor, are indicated. (**D**) Provirus, depicting the Coll.luc reporter gene, used as a sensor to measure AP-1 activity. (**E**) HEK293 cells containing a chromatin-integrated Coll.luc reporter gene were infected with a lentivirus encoding the designer receptor Rαq. In addition, cells were infected with a lentivirus encoding either PLCβ3ct or β-galactosidase (mock). Serum-starved cells were stimulated with CNO (1 μM) for 24 h. Cell extracts were prepared, and luciferase activities and protein concentrations were determined. Luciferase activity was normalized to the protein concentration. Data shown are means +/− SD of four experiments performed in quadruplicate (*** *p* < 0.001).

The aim of this study was to block PLC activity to determine whether this inhibition has an effect on TRP channel signaling. The aminosteroid U73122 has been widely used to inhibit PLCβ enzymatic activity. However, the specificity of this compound is questionable and several PLC-independent effects have been observed [18,19,20,21,22]. Instead, we decided to use genetic inhibition of PLC. The substrate of PLC is a phospholipid embedded in the plasma membrane. PLC must, therefore, bind to the membrane to exert its enzymatic activity. Figure 1A shows the modular structure of the enzyme, including the enzymatic core and the C-terminal regulatory region, as well as the modular structure of a truncated mutant, PLCβ3ct. The C-terminal domain (CTD), encompassing approximately 400 amino acids, is thought to be responsible for maintaining the enzyme in a spatial structure required for enzymatic activation [23]. We expressed the C-terminal domain of PLCβ3, termed PLCβ3ct, together with a FLAG epitope, as depicted in Figure 1B. PLCβ is the major effector enzyme of Gαq-coupled receptors. We tested the activity of the PLCβ3ct mutant in HEK293 cells expressing Rαq (Figure 1C), a designer receptor that specifically couples to Gαq [24]. The designer receptor Rαq is a mutated M3 muscarinic receptor activated with the artificial ligand clozapine-*N*-oxide (CNO) [25,26]. Gαq-coupled receptor signaling can be measured by analyzing the intracellular Ca^2+^ or IP_3_ concentrations, by analyzing the phosphorylation status of ERK1/2, or by analyzing transcriptional activation. We chose to determine transcriptional activation after receptor stimulation, because in this case, a signaling cascade has occurred from the plasma membrane via the cytoplasm into the nucleus. Rαq stimulation has been shown to trigger the activation of the transcription factor AP-1 [24]. We used a collagenase promoter/luciferase reporter gene (Coll.luc, Figure 1D) integrated into the chromatin as a sensor for AP-1 [27] (Figure 1D). Integration into the genome ensured that the reporter gene was embedded into a nucleosomal structure. HEK293 cells were infected with a lentivirus containing the Coll.luc reporter gene and with a lentivirus encoding the designer receptor Rαq. In addition, cells were infected with a lentivirus encoding either PLCβ3ct or β-galactosidase (mock). Figure 1E shows that expression of PLCβ3ct almost completely inhibited Rαq-mediated activation of AP-1. AP-1 activity was reduced by 95.5%. These results indicate that expression of the C-terminal domain of PLCβ3 acts in a dominant-negative manner and prevents signal transduction through Gαq-coupled receptors.

### 2.2. Expression of the C-Terminal Domain of Phospholipase Cβ3 Blocks Phosphorylation of Extracellular Signal-Regulated Protein Kinase (ERK1/2) after Stimulation of a Gαq-Coupled Designer Receptor

**Figure 2:**
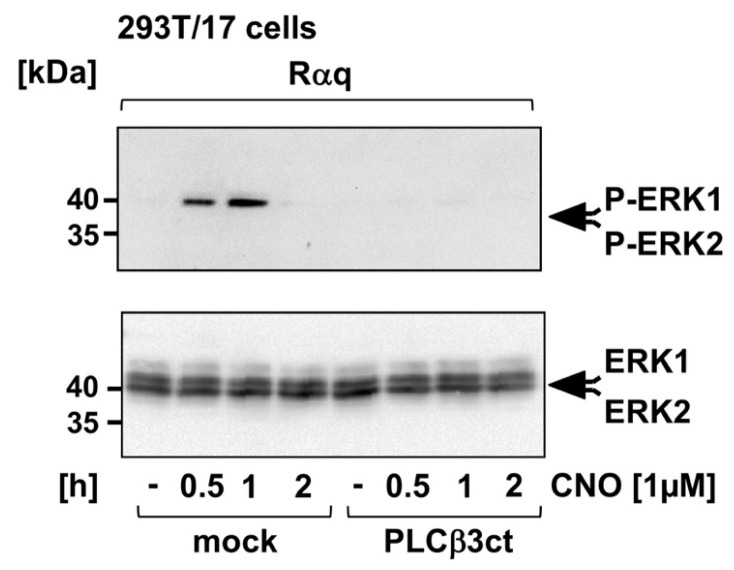
Expression of the C-terminal domain of phospholipase Cβ3 blocks the phosphorylation of extracellular signal-induced protein kinase following stimulation of the Rαq receptor. HEK293 cells expressing the Rαq designer receptor were infected with a lentivirus encoding either PLCβ3ct or β-galactosidase (mock). Cells were serum-starved for 24 h and then stimulated with CNO (1 μM). Cells were harvested at different times. Whole cell extracts were prepared and subjected to Western blot analysis. The blots were incubated with a monoclonal antibody directed against the phosphorylated active form of ERK1/2. As loading control, an antibody detecting ERK1/2 was used.

Stimulation of a Gαq-coupled receptor leads to the phosphorylation and activation of the protein kinase extracellular signal-regulated protein kinase (ERK1/2) [24,28]. Accordingly, the phosphorylation status of ERK1/2 was used to measure Gαq-coupled receptor signaling [29,30]. Figure 2 shows that stimulation of the designer receptor triggered the phosphorylation of ERK1/2 in HEK293 cells. Expression of the PLCβ3ct mutant of PLCβ3 efficiently blocked ERK1/2 phosphorylation and, thus, ERK1/2 activation.

### 2.3. Expression of Membrane-Tagged Cyan Fluorescence Protein Containing the Helix-Turn-Helix Motif (Hα1—Hα2) of PLCβ3 Has No Effect on Downstream Gαq Signaling

**Figure 3:**
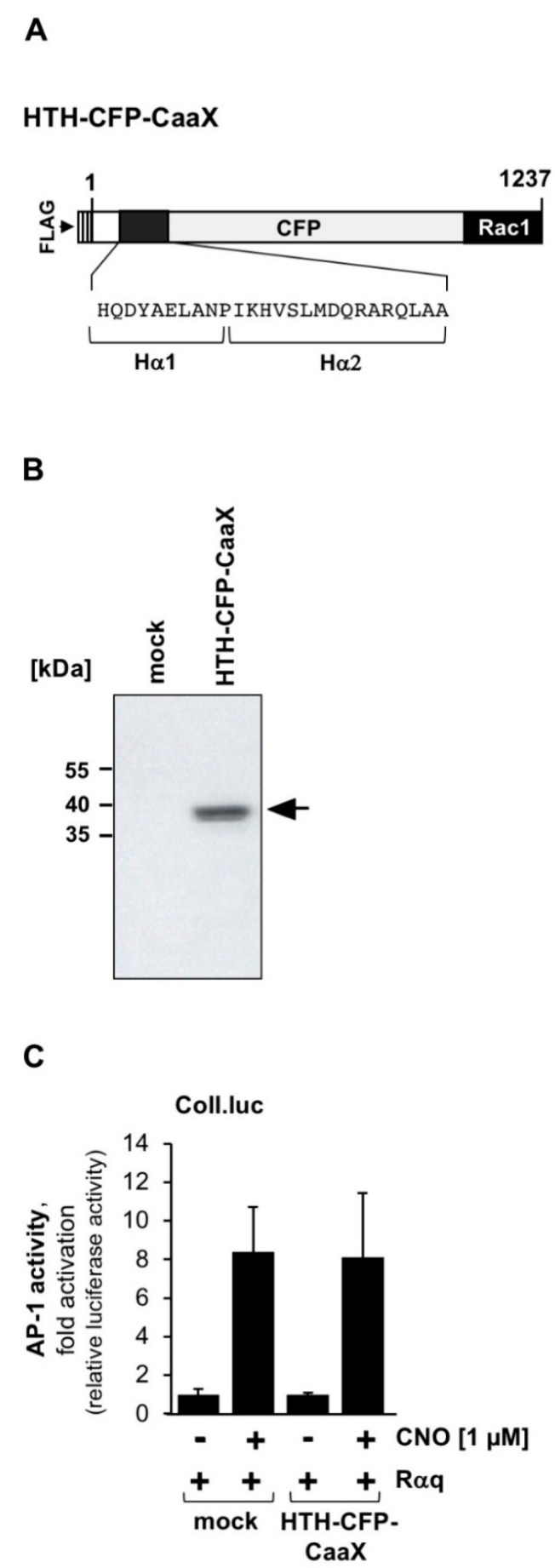
A fusion protein containing the helix-turn-helix (Hα1—Hα2) motif of PLCβ3 does not function as a dominant negative of Gαq-coupled receptor signaling. (**A**) Modular structure of HTH-CFP-CaaX. The FLAG-tagged fusion protein consists of the Hα1—Hα2 domain of PLCβ3, CFP, and the C-terminal domain of Rac1. (**B**) Expression of HTH-CFP-CaaX in HEK293 cells following infection with lentivirus encoding HTH-CFP-Caax. As a control, a virus encoding β-galactosidase (mock) was used for infection. The Western blot was developed with an antibody against the FLAG-tag. kDa, molecular mass markers. (**C**) HEK293 cells containing a chromatin-integrated Coll.luc reporter gene were infected with a lentivirus encoding the designer receptor Rαq. In addition, cells were infected with a lentivirus encoding either HTH-CFP-CaaX or β-galactosidase (mock). Cells were stimulated, harvested, and analyzed as described in the legend to Figure 1 (n = 4). Expression of HTH-CFP-CaaX did not significantly impair Gαq-coupled receptor signaling.

PLCβ enzymes have a helix-turn-helix (HTH) domain C-terminal of the C2 domain, which has been shown to to be an important binding site for activated Gαq [31]. A peptide comprising this HTH motif has been shown to inhibit Gαq-induced activation of PLCβ3 [32], suggesting that it acts as a dominant negative for PLCβ3. We expressed a FLAG-tagged fusion protein, HTH-CFP-CaaX, in HEK293 cells consisting of the HTH domain, a cyan fluorescence protein (CFP), and the C-terminal membrane anchoring domain of Rac1 (Figure 3A). The FLAG-tag was used to show that the fusion protein was expressed as expected (Figure 3B). Next, we tested whether expression of the HTH-CFP-CaaX fusion protein affected Gαq-coupled receptor signaling in HEK293 cells. The results show that there was no significant impairement of Gαq-coupled receptor signal transduction in the presence of the HTH-CFP-CaaX protein, suggesting that expression of the HTH domain of PLCβ3 alone does not inhibit PLCβ signaling.

### 2.4. Expression of the C-Terminal Domain of Phospholipase Cβ1 Blocks Signaling Induced by Stimulation of a Gαq-Coupled Designer Receptor

**Figure 4:**
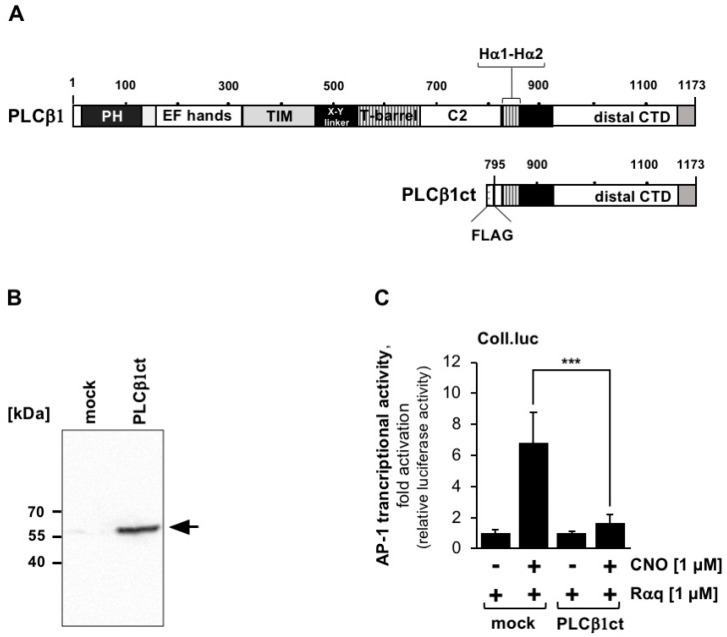
Expression of the C-terminal domain of phospholipase Cβ1 blocks signaling Rαq, a Gαq-coupled designer receptor. (**A**) Multi-domain structure of phospholipase Cβ1 and the truncated mutant PLCβ1ct. PLCβ1 has a similar domain structure as depicted for PLCβ3 in Figure 1. (**B**) Expression verification of PLCβ1ct in HEK293 cells following infection with an PLCβ1ct-encoding lentivirus. Cells were infected with a lentivirus encoding either PLCβ3ct or β-galactosidase (mock). The Western blot was developed with an antibody against the FLAG-tag. kDa, molecular mass markers. (**C**) HEK293 cells containing a chromatin-integrated Coll.luc reporter gene were infected with a lentivirus encoding the designer receptor Rαq. Cells were infected with a lentivirus encoding either PLCβ1ct or β-galactosidase (mock). Serum-starved cells were stimulated with CNO (1 μM) for 24 h. Cells were harvested and analyzed as described in the legend to Figure 1 (n = 3; *** *p* < 0.001).

The PLCβ isoenzymes PLCβ1 and PLCβ4 have been found to be associated with the plasma membrane, whereas the isoenzymes PLCβ2 and PLCβ3 have been found in the cytoplasm [33]. This difference in cellular expression pattern is attributed to sequence differences within the C-terminal domain, which has only 30–35% identical amino acids between the different isoforms [34,35]. Subcellular analysis of GFP-tagged C-terminal domains of PLCβ1 and PLCβ3 revealed that the fusion protein of GFP together with the C-terminal domain of PLCβ1 was clearly visible at the plasma membrane. The fusion protein of GFP together with C-terminal domain of PLCβ3 was also located at the plasma membrane as well as in the cytosol [33]. We, therefore, tested whether the C-terminal domain of the membrane-associated PLCβ1 had similar or even greater activity in inhibiting Gαq-coupled receptor signaling. Figure 4A shows the domain structure of PLCβ1 and the truncated mutant PLCβ1ct. The expression of the mutant in HEK293 cells is depicted in Figure 4B. Cells containing the Coll.luc reporter gene were infected with a lentivirus encoding Rαq. In addition, we infected the cells with a lentivirus encoding either PLCβ1ct or β-galactosidase (mock). The results show that expression of PLCβ1ct was very effective in blocking Gαq-coupled receptor signaling, as underscored by the fact that AP-1 transcriptional activity was reduced by 90.5% in the presence of PLCβ1ct. Thus, no major differences were obtained after expression of the C-terminal domains of PLCβ1 and PLCβ3.

### 2.5. Expression of the C-Terminal Domain of Phospholipase Cβ3 Blocks Intracellular Signaling Triggered by Transient Receptor Potential (TRP) M8 Channel Stimulation

**Figure 5:**
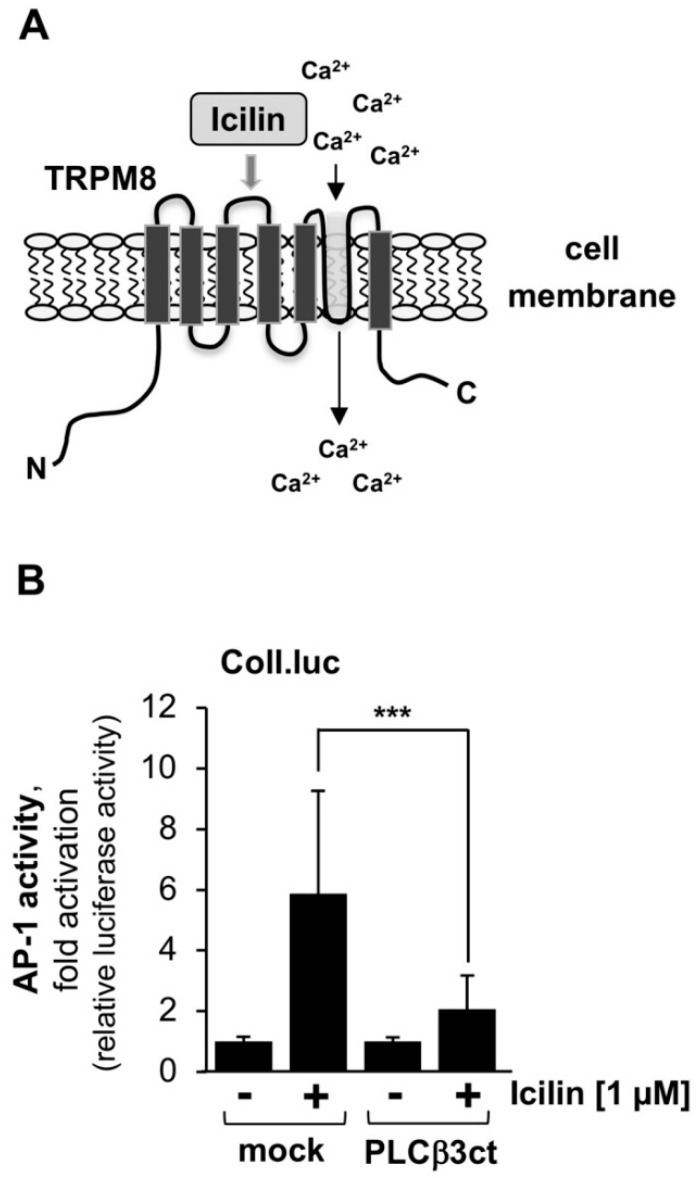
Expression of the C-terminal domain of phospholipase Cβ3 blocks icilin-induced signaling via TRPM8. (**A**) Modular structure of TRPM8. (**B**) HEK293-TRPM8 cells containing a chromatin-integrated Coll.luc reporter gene were infected with a lentivirus encoding either PLCβ3ct or β-galactosidase (mock). Serum-starved cells were stimulated with icilin (1 μM) for 24 h. Cells were harvested and analyzed as described in the legend to Figure 1 (n = 3; *** *p* < 0.001).

Given the hypothesis that many TRP channels “are either activated downstream of the PLC pathway, or modulated by it” [36], we tested whether inhibition of PLCβ3 affects intracellular signaling mediated by TRPM8 channels. These channels are activated by cold temperatures and chemically by cooling agents such as menthol and icilin. Figure 5A shows the modular structure of TRPM8, including the ion pore. Stimulation of TRPM8 channels has been shown to trigger activation of the transcription factor AP-1 [37]. Therefore, we determined the activity of AP-1 as a measure of TRPM8 signaling. HEK293-M8 cells containing an integrated Coll.luc reporter gene were infected with a lentivirus encoding either PLCβ3ct or β-galactosidase. Cells were stimulated with the super-cooling compound icilin to activate TRPM8. Figure 5B shows that TRPM8 signaling was significantly impaired in the presence of PLCβ3ct. Quantification revealed that inhibition of PLCβ3 resulted in 73% inhibition of TRPM8 signaling.

### 2.6. Expression of the C-Terminal Domain of Phospholipase Cβ3 Blocks Intracellular Signaling Induced by Stimulation of TRPM3 Channels

**Figure 6:**
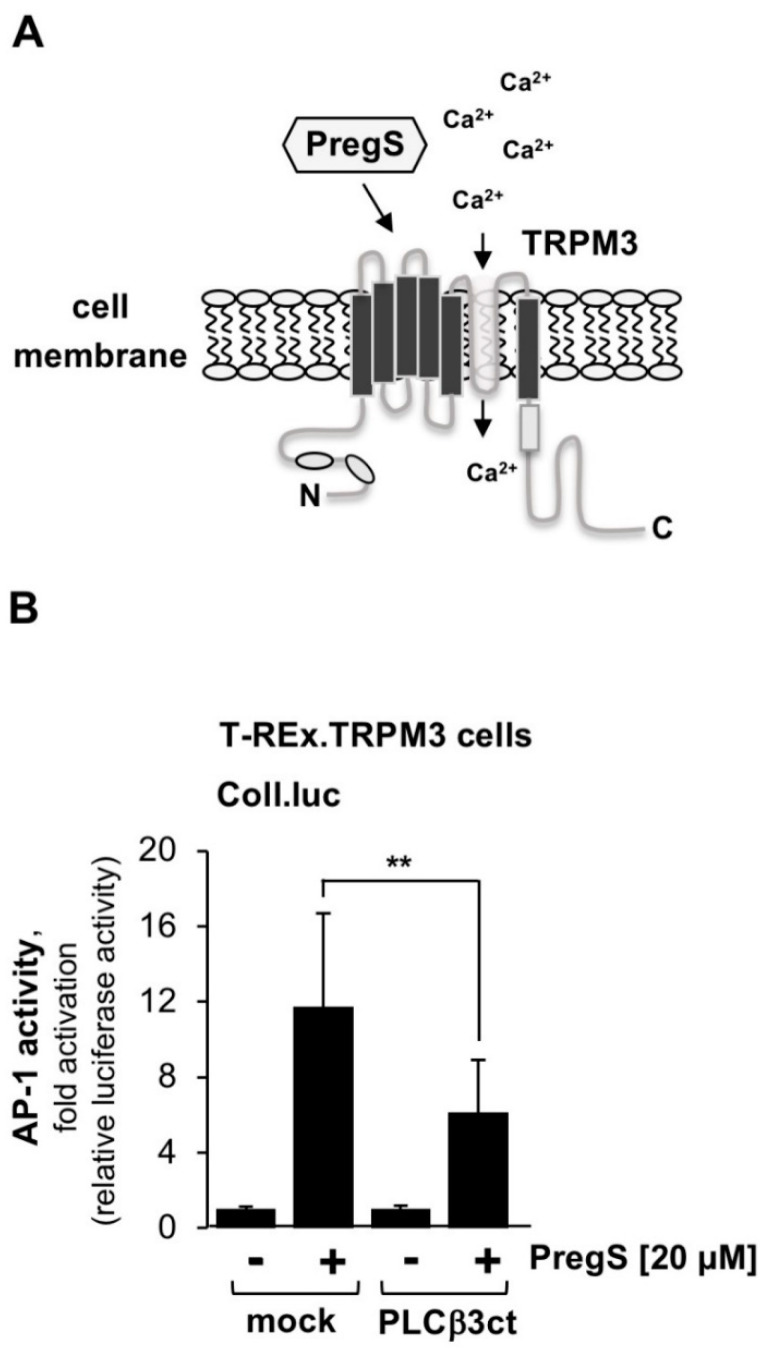
Expression of the C-terminal domain of phospholipase Cβ3 blocks pregnenolone sulfate-induced signaling via TRPM3. (**A**) Modular structure of TRPM3. (**B**) T-REx-TRPM3 cells containing a chromatin-integrated Coll.luc reporter gene were infected with a lentivirus encoding either PLCβ3ct or β-galactosidase (mock). Cells were serum-starved for 24 h in the presence of tetracycline (1 μg/mL) to induce TRPM3 expression. Serum-starved cells were stimulated with icilin (1 μM) for 24 h. Cells were harvested and analyzed as described in the legend to Figure 1 (n = 4; ** *p* < 0.01).

TRPM3 channels are involved in thermoregulation and several other activities, including regulation of insulin secretion. Figure 6A shows the modular structure of TRPM3 channels that can be stimulated with the steroid pregnenolone sulfate. We examined the effect of inhibition of PLCβ3 on TRPM3 signaling. Stimulation of TRPM3 channels triggers the activation of the transcription factor AP-1 [9,10]. Therefore, we determined the activity of AP-1 as a measure of TRPM3 signaling. Figure 6B shows that inhibition of PLCβ3 caused a 46% inhibition of TRPM3 signaling. From these data, we conclude that both TRPM3 and TRPM8 activities are regulated by PLCβ.

### 2.7. The Benzenesulfonamide m-3M3FBS Blocks TRPM3 and TRPM8 Signaling

**Figure 7:**
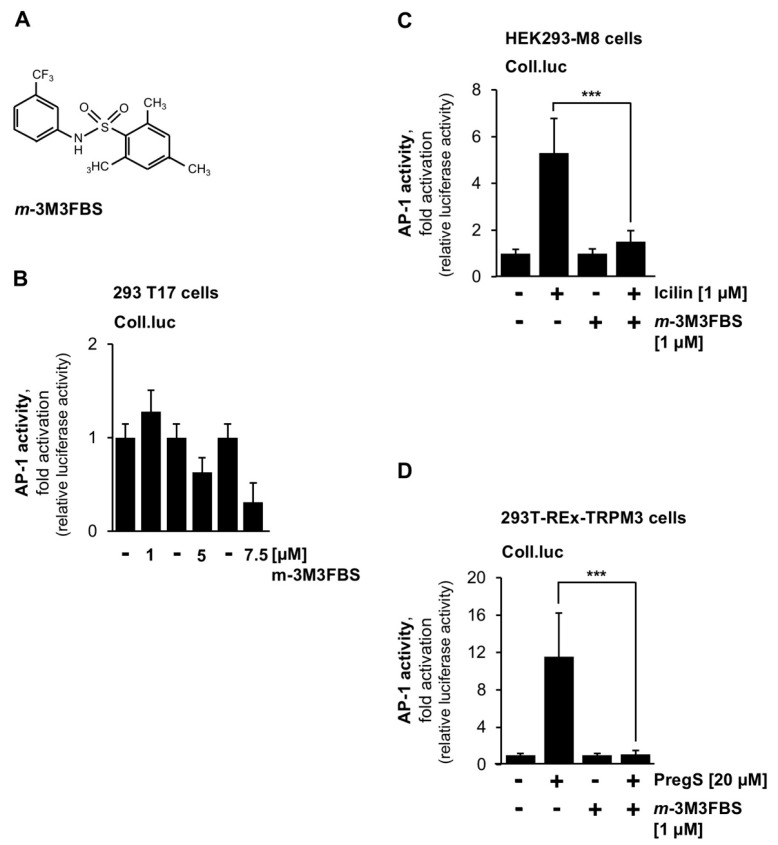
The compound *m*-3M3FBS blocks intracellular signaling mediated by TRPM8 and TRPM3. (**A**) Chemical structure of *m*-3M3FBS. (**B**) HEK293 cells containing the Coll.luc reporter gene were incubated with different concentrations of *m*-3M3FBS for 24 h. Cells were harvested and analyzed as described in the legend to Figure 1 (n = 3). (**C**) HEK293-M8 cells were infected with a recombinant lentivirus containing the Coll.luc reporter gene. Cells were serum-starved for 24 h, preincubated for 3 h with *m*-3M3FBS (5 μM), and then stimulated with icilin (1 μM). Cells were harvested and analyzed as described in the legend to Figure 1 (n = 3; *** *p* < 0.001). (**D**) T-REx-TRPM3 cells were infected with a recombinant lentivirus containing the collagenase promoter/luciferase reporter gene (Coll.luc). Cells were serum-starved for 24 h in the presence of tetracycline (1 μg/mL) to induce TRPM3 expression. Cells were preincubated for 3 h with *m*-3M3FBS (5 μM) and then stimulated with pregnenolone sulfate (PregS, 20 μM) for 24 h in the presence of the inhibitor. Cells were harvested and analyzed as described in the legend to Figure 1 (n = 3; *** *p* < 0.001).

Next, we investigated whether activation of PLCβ affects TRP channel signaling. We used the compound *m*-3M3FBS, which has been described as an activator of PLC [38]. The chemical structure of *m*-3M3FBS is shown in Figure 7A. We hypothesized that pharmacological activation of PLCβ would directly trigger synthesis of IP_3_, release of Ca^2+^ from the endoplasmic reticulum, activation of protein kinase C and ERK1/2, and, subsequently, activation of AP-1 in the nucleus. We tested different concentrations of *m*-3M3FBS for their activity in triggering a signaling cascade leading to AP-1 activation. Figure 7B shows that a concentration of 1 μM marginally activated AP-1 activity, whereas higher concentrations decreased AP-1 activity. On the basis on these experiments, *m*-3M3FBS cannot be considered a PLC activator. Nevertheless, we tested whether *m*-3M3FBS had an effect on TRP channel signaling. Figure 7C,D show that this compound strongly affected TRPM8 (Figure 7C) and TRPM3 (Figure 7D) signaling. Quantification of these results showed that *m*-3M3FBS caused nearly 90% inhibition of TRPM8 signaling and nearly 98% inhibition of TRPM3 signaling. Thus, we conclude that *m*-3M3FBS does not activate PLC but acts as an inhibitor of both TRPM8 and TRPM3 signaling.

### 2.8. The Compound m-3M3FBS Has No Effect on B-Raf-Induced Signal Transduction

**Figure 8:**
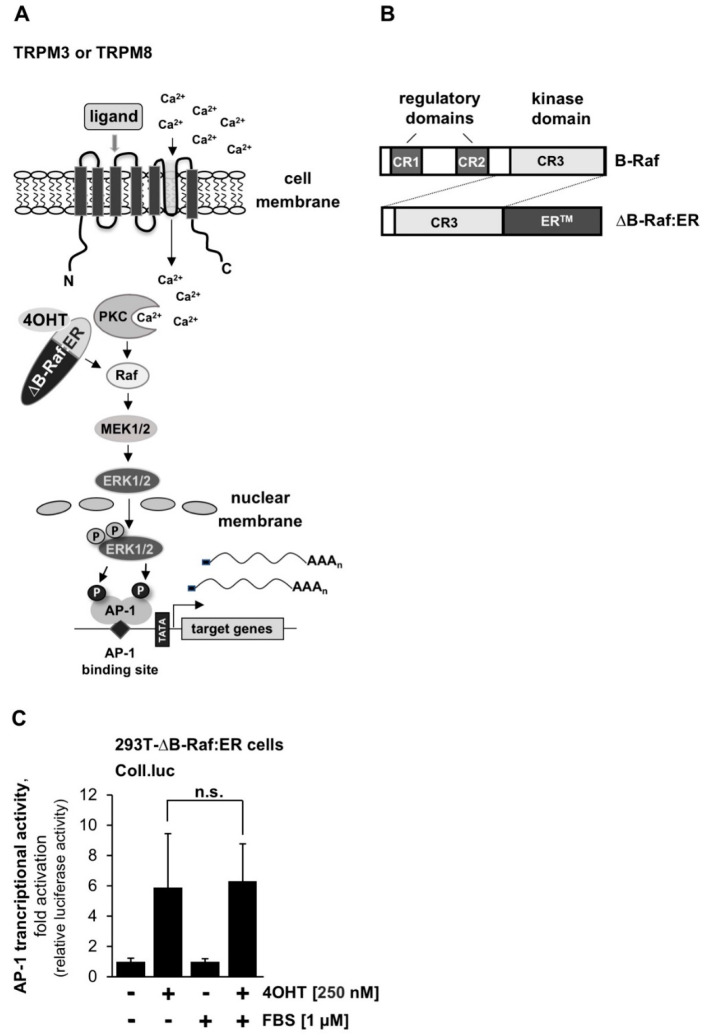
The compound *m*-3M3FBS fails to block B-Raf signaling. (**A**) Signaling pathway connecting TRPM8 and TRPM3 stimulation with AP-1-induced gene transcription. The activation point of the fusion protein ∆B-Raf:ER is indicated. (**B**) Signaling pathway connecting TRPM8 and TRPM3 stimulation with AP-1-induced gene transcription. The activation point of the fusion protein ∆B-Raf:ER is indicated. (**C**) HEK293-∆B-Raf:ER cells were infected with a recombinant lentivirus containing Coll.luc reporter gene. The cells were incubated in medium containing 0.05% serum for 24 h. Stimulation was performed with 4OHT (100 nM) for 24 h. Cells were harvested and analyzed as described in the legend to Figure 1 (n = 3; n.s., not significant).

To locate the target of *m*-3M3FBS, we examined signal transduction of a constitutively active B-Raf protein kinase expressed as a fusion protein together with the estrogen receptor ligand binding domain (Figure 8A). Activation of this B-Raf estrogen receptor fusion protein with the compound 4-hydroxytamoxifen (4OHT) triggers the activation of the ERK1/2 signaling pathway, which ultimately leads to activation of AP-1 in the cells (Figure 8B). Figure 8C shows that the B-Raf-induced activation of AP-1 was not affected in the presence of *m*-3M3FBS, suggesting that this compound alters the activities of targets upstream of B-Raf protein kinase.

## 3. Discussion

The background of this study was the hypothesis that manipulation of PLCβ activity affects TRP channel signaling based on the hypothesis that many TRP channels “are either activated downstream of the PLC pathway, or modulated by it” [36]. Many studies have used pharmacological inhibition of PLC with the compound U73122. However, numerous off-target effects of U73122 have been reported, including activation of TRPM4 channels, inhibition of K^+^ channels, and a PLC-independent release of Ca^2+^ ions from internal stores [16,17,18,19,20,21,22]. U73122 has been reported to induce alkylation of cysteine residues, which may explain its pleiotropic effects. We, therefore, decided to use genetics to inhibit PLCβ.

The C-terminal domain of PLCβ enzymes, comprising approximately 400 amino acids, consists of a proximal and a distal C-terminal domain. The proximal domain, located at the C-terminal of the C2 domain, contains a helix-turn-helix motif (Hα1—Hα2), the primary binding site for Gαq [31], followed by the Hα2’ helix, which has an autoinhibitory activity produced by docking to a cleft near the active site of PLCβ in the absence of Gαq [39]. It has been proposed that the Hα2´ helix prevents the membrane interaction of the PLCβ catalytic core [40]. PLCβ enzymes have to interact with the membrane to efficiently hydrolyze their substrate, phosphatidylinositol 4,5-bisphosphate. The C-terminal domain of PLCβ has been described as a major regulator of membrane binding of the enzymes, as membrane association and PLCβ activity are reduced when this domain is deleted or mutated [41,42,43]. In particular, a coiled-coil structure within the C-terminal domain with clusters of lysine residues is thought to be involved in binding to lipids and/or proteins [44]. Experimentally, it has been shown that the C-terminal domain of PLCβ3 is required to target the enzyme to the lipid monolayer containing phosphatidylinositol 4,5-bisphosphate [23]. Overexpression of the C-terminal domains of PLCβ enzymes impaired the Gαq-mediated increase in intracellular Ca^2+^ concentration and additionally inhibited the hydrolysis of phosphatidylinositol 4,5-bisphosphate [33]. The authors proposed that the C-terminal domain of PLCβ binds to activated Gαq and, in this way, interferes with the interaction of Gαq with wild-type PLCβ enzymes. Expression of the C-terminal domain of PLCβ1 has been shown to block M1 muscarinic acetylcholine receptor-induced activation of α1E Ca^2+^ channels [45]. In this study, we have shown that expression of the C-terminal domain of either PLCβ1 or PLCβ3 efficiently blocked intracellular signaling triggered by stimulation of a Gαq-coupled designer receptor. The expressed C-terminal domains included the proximal C-terminal domain, including the helix-turn-helix motif (Hα1—Hα2), and the extended C-terminal domain, which is thought to be important for the membrane interaction. The Hα1—Hα2 motif has been identified as the major binding site for Gαq, and experiments have shown that a mutated form of PLCγ containing an engineered Hα1—Hα2 domain, becomes subject to regulation by Gαq [31]. We expressed the Hα1—Hα2 domain as a fusion protein together with a fluorescent protein and a membrane anchor and found that this protein was unable to inhibit designer receptor signaling. On the basis of these data, we propose that the expressed C-terminal domains of PLCβ1 and PLCβ3 interfere with plasma membrane components that are necessary for PLCβ biological activity. The most likely candidate, of course, would be phosphatidylinositol 4,5-bisphosphate.

The biological activity of TRP channel activity, including TRPM3 and TRPM8, is regulated by phosphatidylinositol 4,5-bisphosphate. Sophisticated electrogenetic and chemical genetic tools used to reduce plasma membrane phosphatidylinositol 4,5-bisphosphate levels have shown that this phospholipid is required for TRPM3 and TRPM8 channel activities [3,4,6,46]. In addition, it has been suggested that activation of Gαq-coupled receptors, which stimulates PLCβ, inhibits TRPM3 and TRM8 activities [4,6]. Activation of TrkA receptors, which stimulates PLCγ, has been shown to inhibit TRPM8 current [6]. However, a direct relationship between the Gαq-coupled receptor, TrkA or PDGF receptor-induced phosphatidylinositol 4,5-bisphosphate depletion, and impaired TRPM3 and TRPM8 activities was not been presented in this study because the decrease in phosphatidylinositol 4,5-bisphosphate level was estimated only in an indirect manner using the GST-PLCγ-PH biosensor. Due to possible off-target effects, results showing a loss of membrane localization of this biosensor after PLC activation should be taken with caution [16].

In our study, we did not aim to alter the concentration of phosphatidylinositol 4,5-bisphosphate. Rather, we focused on genetically induced inhibition of PLCβ signaling. This study showed that expression of the C-terminal domain of PLCβ3, which contains no enzymatic activity, massively impaired cellular signaling induced by TRPM3 and TRPM8 channels. We hypothesize that the PLCβ3 C-terminal domain masks phosphatidylinositol 4,5-bisphosphate, which is then unavailable for regulation of TRP channel activity.

To complement these data obtained by inhibition of PLCβ, we performed experiments with the compound *m*-3M3FBS, which has been described as an activator of PLC [38]. Several studies have been published directly linking the effects of *m*-3M3FBS to PLC activation and TRP channel activity [47,48]. The results presented in this study show that we were able to archive only a marginal increase in Gαq signaling at a concentration of 1 μM *m*-3M3FBS. Higher concentrations caused inhibition of Gαq signaling and showed increased toxicity. Incubation of the cells with a concentration of 7.5 μM already resulted in detachment of 50% of the cells from their support and we could not investigate whether *m*-3M3FBS acts as an activator of PLC at higher concentrations. Therefore, the results of experiments performed with an *m*-3M3FBS concentration of 25 to 30 μM [47,48,49] are questionable. The classification of *m*-3M3FBS as a PLC activator has also been questioned by others. A study showed that *m*-3M3FBS affects Ca^2+^ homeostasis in a PLC-independent manner [50]. Most importantly, in this study, PLC activity was measured biochemically in the presence and absence of *m*-3M3FBS, in contrast to other reports that used an indirect assay based on translocation of a fluorescently labeled PLCγ-derived PH domain from the plasma membrane to the cytoplasm [47]. This suggests that conclusions based on the use of *m*-3M3FBS as a pharmacological PLC activator should be taken with caution [47,48,49]. In fact, we demonstrated that incubation of the cells with *m*-3M3FBS efficiently inhibited both TRPM3 and TRPM8 signaling. However, the direct target of this compound remains to be identified, and no direct correlation between PLC activity and the effects of *m*-3M3FBS can be postulated.

## 4. Materials and Methods

### 4.1. Cell Culture and Reagents

HEK293T/17 cells were infected with a lentivirus to express of Rαq, a Gαq-coupled designer receptor, as described [24]. HEK293 cells expressing TRP channels TRPM3 (T-REx-TRPM3 cells) and TRPM8 (HEK293-M8 cells) have been described elsewhere [4,51]. HEK293-∆B-Raf:ER cells [52] express a conditionally active B-Raf protein kinase mutant that could be activated with 4-hydroxytamoxifen (4OHT, Sigma # H7904, dissolved in ethanol) for 24 h in medium containing 0.05% fetal calf serum. HEK293 cells, T-REx-TRPM3 cells, HEK293-M8 cells, and HEK293-∆B-Raf:ER were incubated in DMEM containing 0.05% fetal bovine serum for 24 h prior to stimulation. Stimulation was performed with clozapine-*N*-oxide (1 μM CNO, dissolved in ethanol, Enzo Life Sciences, Lörrach, Germany, # NS-105-0005), pregnenolone sulfate (PregS, 20 μM, dissolved in DMSO, Sigma-Aldrich GmbH, Taufkirchen, Germany, # P162), icilin (1 μM, Santa Cruz Biotechnology, Heidelberg, Germany, # sc-201557), or 4-hydroxytamoxifen (4OHT) (100 nM, Sigma # H7904, with ethanol as solvent) for 24 h in medium containing 0.05% fetal bovine serum. Cells were preincubated for 3 h with the compound 2,4,6-Trimethyl-*N*-[3-(trifluoromethyl)phenyl]benzenesulfonamide (*m*-3M3FBS) (Tocris, Bristol, UK, Cat.No. 1941, dissolved in DMSO) at a concentration of 5 μM. Cells were stimulated for 24 h in the presence of *m*-3M3FBS.

### 4.2. Lentiviral Gene Transfer

The lentiviral transfer vector pFUW-Rαq has been described elsewhere [24]. Plasmids pSYFP1-CTbeta1a and pSYFP-CTbeta3, containing the C-terminal domains of PLCβ1 and PLCβ3 fused to YFP, were kindly provided by Joachim Goedhart and Theodorus Gadella, University of Amsterdam, The Netherlands [33]. Plasmid pSYFP1-CTbeta1a was cut with XhoI, filled in with the Klenow fragment of DNA polymerase I, and recut with BamHI. The fragment was cloned into the plasmid 3xFLAG-CMV that had been cut with HindIII, filled in with the Klenow fragment, and recut with BamHI, generating plasmid pCMV-FLAG-PLCβ1ct. This plasmid was cut with Sp1 and EcoRI and filled in with the Klenow fragment of DNA polymerase I. The insert was cloned into a lentiviral transfer vector, generating plasmid pFCW-FLAG-PLCβ1ct. Plasmid pSYFP-CTbeta3 was cut with HindIII, filled in with the Klenow fragment, and recut with BamHI. The fragment was cloned into plasmid 3xFLAG-CMV. This plasmid (pCMV-FLAG-PLCβ3ct) was cut Sp1 and EcoRI, and filled in with the Klenow fragment of DNA polymerase I. The insert was cloned into a lentiviral transfer vector, resulting in plasmid pFCW-FLAG-PLCβ3ct. The FLAG-tagged C-terminal domains of PLCβ1 and PLCβ3 were expressed under the control of the cytomegalovirus IE promoter/enhancer. Plasmid YFP-HTH-CFP-Rac1-CaaX was a kind gift of John Sondek, University of North Carolina, Chapel Hill, NC, USA [32]. The plasmid was cut with BamHI and EcoRI and cloned into the filled in BglII site of plasmid 3xFLAG-CMV, resulting in plasmid pCMV-FLAG-HTH-CFP-Rac1-CaaX. This plasmid was cut with BamHI, filled in with the Klenow fragment of DNA polymerase I, and recut with Ecl136II. The fragment was cloned into HpaI-cut plasmid pFUW, resulting in lentiviral transfer plasmid pFUW-FLAG-HTH-CFP-Rac1CaaX. Viral particles were produced by triple transfection of HEK293-TN cells with the gag-pol-rev packaging plasmid, the pCMVG plasmid that encodes the glycoprotein of vesicular stomatitis virus, and the lentiviral transfer vector [53].

### 4.3. Reporter Gene Assay

The lentiviral transfer vector pFWColl.luc has been described elsewhere [27]. Infected cells were maintained in medium containing 0.05% fetal bovine serum for 24 h and then stimulated with the appropriate ligands for 24 h. Cell extracts were prepared using reporter lysis buffer (Promega, Mannheim, Germany) and assayed for luciferase activities. Luciferase activity was normalized to the protein concentration. Luciferase activities of the extracts were measured using a luminometer (Berthold Detection Systems, Pforzheim, Germany). The light units were normalized to the protein concentration of the extracts, which was determined using a BCA protein assay kit.

### 4.4. Western Blots

Thirty micrograms of proteins were separated by SDS-PAGE. Blots were incubated with an antibody directed against either the extracellular signal-regulated protein kinase ERK1/2 (Santa Cruz Biotechnology, Heidelberg, Germany, # sc-153) or the phosphorylated form of ERK2 (Santa Cruz Biotechnology, Heidelberg, Germany, # sc-7383). The anti-ERK2 antibody reacted to a lesser extent with ERK1. FLAG-tagged proteins were detected in Western blot experiments using monoclonal antibody M2 (Sigma-Aldrich, Steinheim, Germany, # F1804) at a dilution of 1:3000 in TBS. Immunoreactive bands were detected with enhanced chemiluminescence, using a 1:1 solution of solution 1 (100 mM Tris-HCl, pH 8.5, 5.4 mM H_2_O_2_) and solution 2 (2.5 mM Luminol, 400 μM p-coumaric acid, 100 mM Tris-HCl, pH 8.5).

### 4.5. Statistics

Data shown are means +/− SD of at least three independent experiments performed in quadruplicate. The two-tailed Student´s *t*-test was used for the statistical analyses. Statistical probability is expressed as *** *p* < 0.001; ** *p* < 0.01, and * *p* < 0.05. We considered values significant when *p* < 0.05.

## 5. Conclusions

Expression of PLCβ1ct and PLCβ3ct, truncated forms of PLCβ1 and PLCβ3 comprising the C-terminal membrane association domains of the enzymes, almost completely blocked the signal transduction of a Gαq-coupled designer receptor. Moreover, expression of PLCβ3ct significantly impaired signal transduction of TRP channels TRPM3 and TRPM8. The C-terminal domain of PLCβ3 interacts with plasma membrane targets, most likely phosphatidylinositol 4,5-bisphosphate, and, in this way, blocks the biological activation of TRPM3 and TRPM8, which require interaction with this phospholipid. Thus, PLCβ regulates TRPM channels by masking phosphatidylinositol 4,5-bisphosphate with its C-terminal domain.
